# Optical and MRI Multimodal Tracing of Stem Cells In Vivo

**DOI:** 10.1155/2023/4223485

**Published:** 2023-12-19

**Authors:** Jia Yang, Min Yan, Zhong Wang, Cong Zhang, Miao Guan, Zhenglong Sun

**Affiliations:** ^1^State Key Laboratory of Primate Biomedical Research, Institute of Primate Translational Medicine, Kunming University of Science and Technology, Kunming, Yunnan 650500, China; ^2^Yunnan Key Laboratory of Primate Biomedical Research, Kunming, Yunnan 650500, China; ^3^Affiliated Mental Health Center of Kunming Medical University, Kunming, Yunnan 650000, China; ^4^Faculty of Life Science and Technology, Kunming University of Science and Technology, Kunming, Yunnan 650500, China

## Abstract

Stem cell therapy has shown great clinical potential in oncology, injury, inflammation, and cardiovascular disease. However, due to the technical limitations of the in vivo visualization of transplanted stem cells, the therapeutic mechanisms and biosafety of stem cells in vivo are poorly defined, which limits the speed of clinical translation. The commonly used methods for the in vivo tracing of stem cells currently include optical imaging, magnetic resonance imaging (MRI), and nuclear medicine imaging. However, nuclear medicine imaging involves radioactive materials, MRI has low resolution at the cellular level, and optical imaging has poor tissue penetration in vivo. It is difficult for a single imaging method to simultaneously achieve the high penetration, high resolution, and noninvasiveness needed for in vivo imaging. However, multimodal imaging combines the advantages of different imaging modalities to determine the fate of stem cells in vivo in a multidimensional way. This review provides an overview of various multimodal imaging technologies and labeling methods commonly used for tracing stem cells, including optical imaging, MRI, and the combination of the two, while explaining the principles involved, comparing the advantages and disadvantages of different combination schemes, and discussing the challenges and prospects of human stem cell tracking techniques.

## 1. Introduction

In recent years, an increasing number of scientific studies have shown that stem cells have unique biological properties and functions, including multidirectional differentiation potential, promotion of tissue regeneration, secretion of anti-inflammatory and nutritional factors [1–4], regulation of tissue microenvironment [5, 6], immunomodulation, promotion of restoration of immune system homeostasis [4, 7–9], and homing. Stem cell therapy has shown great clinical potential in, for example, tumors, injury, inflammation, and cardiovascular diseases [3, 9–15]. However, numerous stem cell therapies remain in the preclinical stage, mainly due to the uncertain safety of stem cells and the necessity of evaluating treatment effectiveness and tracking cell behavior in vivo. Scientists need real-time, unbiased, long-term tracking of stem cell homing, differentiation, proliferation, and survival in vivo. Therefore, stem cell monitoring plays a crucial role in ensuring the overall success and improvement of stem cell-based therapies.

The commonly used stem cell tracing methods include optical, magnetic resonance imaging (MRI), and nuclear medicine imaging [16, 17]. Nuclear medicine imaging and MRI are commonly used in clinical practice for tracing cells. For instance, ^111^In-oxiquinolon, a radioactive label approved by the FDA [16], is used to monitor cell migration. However, as this label emits radiation, there are potential safety risks when monitoring transplanted cells over the long term. Additionally, nuclear medicine imaging has limited spatial resolution, which can lead to insufficient accuracy in the positioning of cells. In contrast, MRI is a noninvasive imaging modality with high spatial resolution, making it a popular choice in clinical settings for monitoring magnetically labeled cells. The first report of using MRI to track therapeutic cells in humans dates back to 2005, and MRI was proven at that time to be at least as sensitive as nuclear medicine imaging with better spatial resolution [18].

However, MRI has low resolution at the cellular level, and optical imaging has poor tissue penetration in vivo. It is difficult for any single imaging method to simultaneously meet the requirements of noninvasiveness, high penetration, and high resolution, so the information obtained by any single imaging modality is relatively limited and one-sided.

Multimodal imaging combines different imaging modalities to obtain information about multiple parameters simultaneously. Since different imaging methods have different imaging principles and acquire different information, by combining the advantages of different imaging methods through multimodal imaging, complementary and comprehensive information can be obtained. Multimodal imaging has been increasingly used to track transplanted stem cells in vivo. Multimodal optical imaging can obtain information on the physiological activity process of transplanted stem cells in vivo [19–22]. In addition to providing high-resolution information about tissue anatomy, multimodal MRI can also accurately locate and quantify transplanted stem cells in deep tissues [23, 24]. The multimodal imaging system combined with optical and MRI can present functional and structural imaging to achieve multidimensional visualization of the localization, differentiation, and survival status of transplanted stem cells in vivo, which can facilitate the determination of stem cell fate in vivo in a more realistic and comprehensive manner.

**Figure 1:**
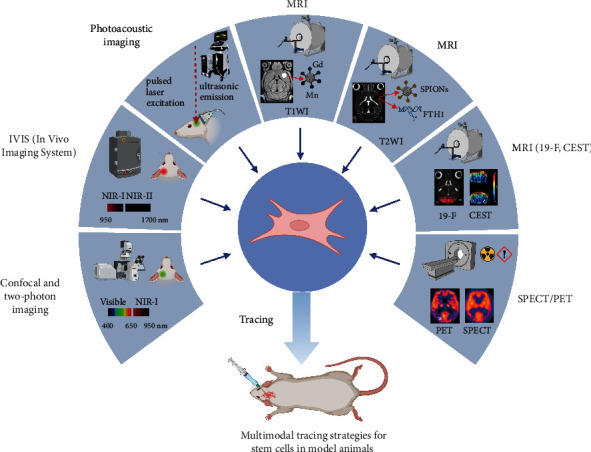
Multimodal tracing strategies for stem cells in model animals.

This review provides an overview of various multimodal imaging technologies and labeling methods commonly used for tracing stem cells, including optical imaging, MRI, and the combination of the two, while explaining the principles involved, comparing the advantages and disadvantages of different combination schemes, and discussing the challenges and prospects of human stem cell tracing technology (Figure 1).

## 2. Main Body

### 2.1. Labeling Methods

Whether performed by optical imaging or MRI, the imaging of transplanted cells in vivo often requires the cells to be labeled, and imaging systems determine cell activity by identifying tags that are easy to detect in the body. Commonly used labeling methods can be divided into direct labeling and indirect labeling [16].

**Figure 2:**
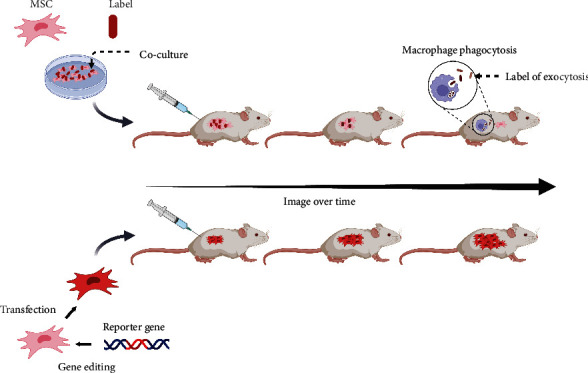
Characteristics of different labeling methods for cell tracing in vivo.

In direct labeling, cells and imaging labels are coincubated. Their combination mainly relies on electrostatic interactions, and direct labeling is convenient and safe to use. However, the signal of the label dilutes with time and cell division, while the phagocytosis of dead labeled cells by macrophages or exocytosis of intracellular labels can produce false positives, which greatly limits the time available for cell tracing (Figure 2).

Indirect labeling integrates reporter genes into cells for stable expression through gene editing. Although the operation is complex and time-consuming, as the cells proliferate, the reporter genes will be passed on to the daughter cells and observed throughout the life cycle of the cells to evaluate cell proliferation in vivo [16, 25] (Figure 2).

### 2.2. Optical Methods

Bio-optical imaging is an important tool for biomedical research on stem cells in vivo, visualizing physiological and pathological activities at the molecular and cellular levels with higher sensitivity and specificity than other imaging techniques. Optical imaging includes different imaging principles: fluorescence imaging, bioluminescence imaging (BLI), photoacoustic imaging, and optical coherence tomography (OCT). Different optical imaging techniques have different advantages and limitations for in vivo stem cell tracing.

#### 2.2.1. Fluorescence Imaging

Fluorescence imaging techniques use fluorescent dyes or reporter genes to label stem cells and generate light signals that are then detected by optical detection instruments under external excitation. Confocal and two-photon imaging has absolute advantages in assessing the functions of transplanted stem cells, such as cell proliferation, differentiation, and migration. For example, Turcotte et al. traced green fluorescent protein- (GFP-) labeled individual stem cells and observed multiple GFP-labeled cells in the bone marrow lumen on day 5, providing visual evidence of local proliferation of individual transplanted stem cells [26]. Teo et al. observed the migration and aggregation of fluorescent dye-labeled mesenchymal stem cells (MSCs) from intravascular to inflammatory sites by confocal microscopy [27]. However, the uneven thickness of living tissues and the absorption and scattering of visible light in tissues limit the penetration of fluorescence imaging, so the application of fluorescence imaging technology for in vivo tracing is limited to specific parts of the body, such as the designated bone marrow cavity within 100 *μ*m below the surface of the skull of living mice [26] and the skin of mouse ears [27].

Near-infrared fluorescence (NIR) has a small scattering rate and weak light absorption within the tissue, which improves the depth of imaging, and is accordingly relatively widely used in in vivo tracing. In particular, the second near-infrared window (NIR-II, 1000-1700 nm) exhibits better tissue penetration and spatiotemporal resolution than the first near-infrared window (NIR-I, 700-900 nm), which provides a great improvement in fluorescence imaging-based stem cell tracing technologies [28–30].

With the development of fluorescent nanomaterials, an increasing number of durable, stable, and safe nanoprobes are now being used for stem cell tracking and therapy [31, 32]. In addition, the ease of design of nanomaterials plays a great role in revealing the activity and function of stem cells. For example, stem cell death in vivo generates excessive reactive oxygen species (ROS), and the ability of stem cells to survive in vivo can be precisely quantified by constructing ROS-sensitive fluorescent probes [33, 34]. The dual-channel NIR imaging system synthesized by Kim et al. was able to track the behavior of stem cell-mediated bone regeneration and its relationship with scaffold degradation in vivo [35].

#### 2.2.2. Bioluminescence Imaging (BLI)

Unlike fluorescence imaging, BLI does not require external excitation light to produce light signals; instead, reporter genes are used to label cells or DNA with self-luminescence. The most common bioluminescent reporter is luciferase. In living organisms, luciferase undergoes a biochemical reaction with luciferin to produce a light signal. Due to the lack of self-luminescence, bioluminescence produces a high signal-to-noise ratio with good sensitivity and specificity at the molecular level. The bioluminescence reaction requires the involvement of many cofactors, which can indicate the activity of the labeled cell. Based on the above advantages, BLI is widely used for tracing transplanted cells in small animals [36, 37].

#### 2.2.3. Photoacoustic Imaging

Photoacoustic imaging is a new noninvasive and nonionizing biomedical imaging method that has been developed in recent years. This technique works by shining a pulsed laser light into biological tissue, which then absorbs the light energy and generates ultrasonic waves due to thermoelastic expansion. These ultrasonic waves can be detected by a specialized detector array, allowing the three-dimensional imaging of biological tissues. Photoacoustic imaging combines the advantages of optical and ultrasound imaging to provide high-resolution, high-contrast images at deeper imaging depths. In addition, stem cells can be more accurately localized to the transplantation site under real-time ultrasound guidance [38].

### 2.3. MRI Methods

MRI is a noninvasive, radiation-free imaging modality with high anatomical resolution that has no limitations on imaging depth and enables the whole-body scanning of living organisms. Conventional MRI generates signals through the excitation of water (H) protons (the most abundant in the body) by applied radio frequency pulses in a constant magnetic field; resonance occurs, and reconstructed images are obtained through signal acquisition and computer processing.

MRI contrast agents and reporter genes do not directly show MRI signals, but by changing the relaxation time of H protons within cells, MRI signals are produced that are different from those of host cells, and thus, transplanted cells can be imaged. MRI is a multiparametric imaging modality that produces different weighted images, and the contrast agent and reporter genes required to label cells in each weighted image are also different. Common MRI-weighted images include longitudinal relaxation-weighted imaging (T1WI) and transverse relaxation-weighted imaging (T2WI and T2^∗^WI). T2^∗^ is a type of gradient recalled echo sequence that lacks a 180° radiofrequency pulse to refocus, making it subject to the susceptibility effect.

#### 2.3.1. T2WI-Based Tracing

On T2WI or T2^∗^WI, the T2 contrast agent shortens the transverse relaxation time of surrounding water protons, resulting in a decrease in signal intensity, thus producing a dark image. At present, superparamagnetic iron oxide nanoparticles (SPIONs) are widely used, and they are considered relatively stable and safe T2 contrast agents compared to other contrast agents. Their basic structure consists of an iron oxide core and surface coating. Iron is essential for the human body to maintain normal function, so the dose of injected SPIONs is considered to be within the safe range of human tolerance, and the coating can prevent nanoparticle aggregation, improve biocompatibility, and reduce toxicity [39, 40].

Common MRI reporters include the following: genes encoding enzymes, such as *β*-galactosidase expressed by the LacZ reporter gene, to promote contact between water ions and metal ions [41]; supracellular receptor genes, such as Timd2 [42], a receptor that mediates ferritin endocytosis; transferrin receptor 1 (TfR1) [43, 44], which increases iron uptake; and endogenous reporter genes, such as ferritin heavy chain reporter gene (FTH1) [45]. Most of the common MRI reporter genes are T2WI reporter genes. Tyrosinase reporter genes express tyrosinase, which promotes melanogenesis, and melanin binding to metal ions (Fe^3+^) can alter T1 and T2 relaxation times while increasing T1WI and T2WI image contrast [46]. However, the introduction of reporter genes increases biological safety risks, such as the risk of disrupting iron homeostasis in vivo [44]. To minimize potential risks, He et al. introduced the Tet-On gene switch to regulate Fth1 expression according to the requirements of MRI [47]. Sun et al. added the promoter of the tumor-specific gene PEG3 upstream of the reporter gene FTH1, and when the expression of FTH1 increased significantly after stem cells were induced for malignant transformation, the MRI monitoring system could help detect the malignant transformation of transplanted stem cells at an early stage [48].

In either the SPION or FTHI reporter gene marker method, the contrast of dark signals generated by T2WI is relatively insensitive and not easy to identify by the naked eye [44], especially in the presence of bleeding, calcification, and metal deposition. When using T2WI sequences to track cells, it is often necessary to provide prior information to verify and supplement their results.

#### 2.3.2. T1WI-Based Tracing

With T1WI-based tracing, the T1 contrast agents increase the signal intensity by shortening the longitudinal relaxation time of protons, resulting in bright images, which are generally represented by gadolinium- (Gd^3+^-) and manganese- (Mn^2+^-) based contrast agents. Manganese (Mn), as a magnetic substance, is a potential T1 contrast agent [49, 50]. Szulc et al. combined Mn with cells overexpressing the ferritin reporter gene to produce highly efficient bright contrast at T1WI, with significantly higher sensitivity for live cell monitoring than the dark contrast produced by conventional binding to iron [51].

In addition to providing anatomical information, manganese-related MRI contrast agents can also provide functional information. The unique ability of Mn^2+^ to enter metabolically active living cells via voltage-gated calcium channels increases the local contrast in T1WI images, thus allowing the assessment of stem cell function in vivo, as shown by Jiang et al., who monitored neural activity induced by induced pluripotent stem cell-derived neural stem cells in local brain regions, and Kim et al., who evaluated transplanted stem cells for cardiomyocyte therapy [52–54].

Gadolinium-based chelators, widely used as clinical T1 contrast agents, currently serve as nonspecific contrast agents in the extracellular space. Despite efforts like optimizing surface modification and hypo-osmotic labeling to enhance stem cell labeling efficiency and in vivo imaging time [55–59], numerous studies show that monitoring Gd-labeled cells is typically limited to around 10 days [55, 60]. This restriction poses a challenge for achieving long-term tracking objectives.

#### 2.3.3. ^19^F-Based Tracing

Although the application of T1WI and T2WI contrast agents in conventional MRI (^1^H-MRI) can increase the contrast between the transplanted stem cells and the surrounding tissue, the contrast enhancement is limited in such a strong H proton MRI signal background. Scientists developed fluorine 19-MRI (^19^F-MRI) as a complement to the shortcomings of conventional ^1^H-MRI. F elements are almost absent in the human body (only present in bones and teeth).^19^F is used as a probe to label cells, and probing the signal distribution and image intensity of ^19^F probes introduced into the body yields images without biological background interference and with high specificity [61], which can then be superimposed on conventional ^1^H-MRI images to acquire both ^19^F-MRI and ^1^H-MRI images to achieve a complementary ^1^H-MRI image. In addition, the signal intensity acquired by ^19^F-MRI is linearly proportional to the ^19^F content, allowing the quantification of cell numbers [62].

#### 2.3.4. Chemical Exchange Saturation Transfer- (CEST-) Based Tracing

CEST is a novel MRI technique that enables the detection and quantification of low-concentration molecules in tissues. In CEST, a radiofrequency pulse is applied to selectively saturate the protons of one of the pools of molecules. Then, after a certain time delay, an MRI image is acquired, which shows a decrease in signal intensity in the regions where the protons from the saturated pool have exchanged with those of the unsaturated pool. By measuring the resulting decrease in signal intensity, it is possible to quantify the concentration of the exchanging molecule.

Yuan et al.'s team achieved in vivo tracing of MSCs without additional labeling by mannose-weighted CEST MRI using high-mannose N-linked glycans expressed abundantly on the surface of human bone marrow mesenchymal stem cells (hMSCs) as biomarkers [63]. The absence of chemical labeling avoids the potential risk of direct and indirect labeling operations causing changes in stem cell properties.

### 2.4. Multimodal Imaging

#### 2.4.1. Multimodal Optical Imaging

Multimodal optical imaging can utilize the advantages of multiple optical imaging to achieve stem cell tracing by complementing limitations of fluorescence imaging. For example, Nguyen et al. tracked the migration of transplanted cells in the damaged retinal pigment epithelium (RPE). Through the use of a three-modal optical imaging system combining fluorescence imaging, photoacoustic imaging, and optical coherence tomography, transplanted cells were tracked in the retinal layer for 3 months, while the acquisition of both photoacoustic images and OCT images provided accurate retinal anatomy information [20]. Comenge et al. induced coexpression of the photoacoustic imaging reporter near-infrared fluorescent protein iRFP720 with the BLI reporter luciferase, further prolonging the time photoacoustic imaging could track stem cells in vivo and distinguishing false-positive signals that may be produced by direct labeling [21]. Gold nanomaterials, as photoacoustic imaging contrast agents, have high photothermal conversion efficiency, and thus, photothermal therapy can be performed on tumors at the same time as multimodal imaging. Ning et al. combined gold nanomaterials with fluorescent dyes in a model of stem cell therapy to further inhibit the growth of tumor cells through photothermal therapy [22]. Qi et al. utilized second harmonic generation (SHG) to address the challenge of limited tracing time for stem cells in vivo caused by fluorescence quenching. This approach was applied in a mouse model where transplanted stem cells were used to enhance skin wound healing. They constructed an SHG probe to label GFP-MSCs. In addition to continuing to observe the SHG signal after fluorescence quenching, the SHG imaging technique allows imaging of collagen fibers, skin tissues, and corneal stroma without requiring additional fluorescent dyes [64]. This eliminates the safety hazard of using multiple fluorescent labels to monitor several targets and provides novel ideas for stem cell tracking that can be translated to the clinic.

Moreover, the integration of fluorescence imaging and bioluminescence techniques can provide a multifaceted demonstration of the condition of transplanted stem cells in vivo [65].

Huang et al. utilized NIR-II probes along with endogenous red firefly luciferase- (RFLuc-) based BLI to observe the distribution and activity of transplanted hMSCs in a mouse model of calvarial defects. Collagen type 1 promoter-driven Gaussia luciferase- (GLuc-) based BLI was employed to assess the impact of transplanted hMSCs on osteogenic differentiation, providing insight into the in vivo function of hMSCs [66].

#### 2.4.2. Multimodal MRI

Although MRI is a multiparametric imaging technique, conventionally labeled cells can only be visualized in a single modality of magnetic resonance images. To increase the sensitivity of tracer cells in different MRI sequences, T1-T2 bimodal MRI contrast agents have been proposed and developed. Dual-mode contrast agents can simultaneously provide high signal contrast for T1WI sequences and low signal contrast for T2WI sequences, enabling cross-complementary verification of image information on the same imaging device and avoiding the problem of multimodal image mismatch between different devices [67, 68]. The construction of bimodal contrast agents can be achieved by changing the size of iron oxide nanoparticles [69, 70] and manganese-doped iron oxide nanoparticles [24]. However, the simple combination of the two contrast agents will cause mutual signal interference, which poses a challenge to the accurate localization of cells in the body. Shin et al. developed a nanoparticle imaging agent to achieve T1-T2 dual-mode MRI contrast and artifact filtering to accurately track the migration of neural stem cells to the infarct region in a rat stroke model [68]. At present, the application of T1-T2 bimodal contrast agents in vivo still faces a series of problems, such as stability, cytotoxicity, and metabolism.

Susceptibility-weighted imaging (SWI) involves a three-dimensional sequence with improved spatial resolution and enhanced magnetic sensitivity developed from a simple T2^∗^-weighted two-dimensional sequence that is sensitive to magnetic field inhomogeneity and can sense small changes in the magnetic field. Lei et al. transplanted polyethylene glycol/polyethyleneimine-modified superparamagnetic iron oxide- (PEG/PEI-SPIO-) labeled stem cells into the brain during stem cell therapy of an Alzheimer's disease model [71]. Small magnetic field changes occurred, the contrast of T2^∗^-weighted images was enhanced through the additional use of SWI, and it was thus easy to observe transplanted cells on the basis of T2WI.

#### 2.4.3. Multimodal Optical Imaging and MRI

Optical imaging enables tracing at single-cell resolution but does not allow whole-body scanning due to tissue penetration issues; MRI enables whole-body scanning and provides clear soft tissue anatomy, but imaging resolution at the cellular level is low. The combination of multiple imaging methods and multifunctional probes working in concert can improve the sensitivity of stem cell detection in vivo, the resolution of imaging, and the time of surveillance and provide a comprehensive response to the localization and expression of stem cell therapy in vivo. Multimodal imaging is gradually becoming a preferred option in preclinical experiments for in vivo stem cell tracing, and thus, the optical-MRI modality has great potential in translation from laboratory to clinical applications.

*(1) MRI/Fluorescence Multimodal Imaging*. In dual-modality imaging with combined MRI and fluorescence, constructing a single probe with MRI and fluorescence imaging capabilities is widely used to observe the same target in different imaging modalities. The advantage of a single probe is that it helps ensure that each modality has the same pharmacokinetics and signal colocalization while avoiding the increased safety risks associated with multimodal imaging when multiple doses of contrast agents need to be added.

The most widely used probes are nanomaterials, which are highly controllable and provide large payloads and high-resolution images. For instance, fluorescent dyes are added to silicone layers that allow surface functionalization with iron oxide magnetic nanoparticles (MNPs) as the core, showing high fluorescence intensity as well as contrast of MRI T2WI sequences, increasing the sensitivity and specificity of diagnosis [72]. Feng et al. [73] introduced lanthanide ions in paramagnetic nanoparticles to achieve both magnetic and near-infrared luminescence functions. A significant NIR luminescence signal was observed in the abdominal cavity of mice, showing excellent deep tissue penetration, but when tracking stem cells in the mouse brain, NIR penetration is low in the cranial region, and MRI provides a valuable complement to achieve stem cell tracking in different parts of the body. However, the use of multiple contrast agents may cause the agents to interfere with each other, which can disrupt imaging. The Gd^3+^ and Mn^2+^ codoped CulnS2-ZnS nanoprobes constructed by Chetty et al. effectively control the relative response between multiple dopants while tracking the tumor tropism of stem cells injected intravenously under three imaging modalities of NIR, MRI, and CT, providing a reference for stem cell-assisted anticancer therapy and tracking tissue and organ regeneration [74].

However, multimodal single probes that directly label cells still cannot avoid the limitations of direct labeling, such as short cell monitoring times, identical signal sources, and lack of complementary signals. In addition, MRI has much lower sensitivity than optical imaging, with different concentration requirements for different contrast agents in a single probe. Moreover, although the sensitivity of optical imaging is high, the sensitivity of different imaging sites in vivo will have different degrees of attenuation, and different ratios need to be designed according to the experimental purpose, which are great challenges for the clinical translation of multimodal single probes at this stage.

To monitor the fate of transplanted stem cells in vivo for a long period, the indirect labeling of stem cells is highly desired. For example, stem cells coexpressing the MRI reporter gene FTH1 and enhanced green fluorescent protein (EGFP) were introduced in small animal models of cerebral ischemic stroke and brain tumors, and the tropism of stem cells to intracerebral lesions was observed by MRI, while fluorescent protein validated MRI observations in in vitro fluorescence imaging [75, 76]. Although studies have demonstrated the ability of the MRI reporter gene FTH1 to monitor stem cells in vivo over time, the contrast of FTH1-labeled cells in MRI is limited [44]. Fluorescence imaging complements the information at the cellular level but still provides limited information in vivo.

*(2) MRI/BL Multimodal Imaging*. Bioluminescence imaging is widely used in multimodal imaging techniques because it provides sensitive molecular-level information easily and inexpensively. Nonetheless, bioluminescence imaging has shortcomings in locating specific areas within the body. To overcome this limitation and improve image quality, MRI is frequently employed because it provides valuable cell-related anatomical information.

In a study by Thin et al. to assess the efficiency of stem cell delivery to breast cancer, stem cells carrying bioluminescent reporter genes were incubated with a bimodal imaging probe with magnetic (SPION) and radioactive (indium-111-oxine) properties to form trimodal imaging-capable stem cells. The in vivo BLI signal intensity responds to cell viability [77]. MRI provides excellent spatial resolution and confirms the localization of delivered stem cells, but MRI was unable to accurately quantify cells due to the dephasing effect of SPIONs on surrounding magnetic spins, and SPECT imaging allowed the semiquantitative assessment of cell numbers in vivo. Finally, histological analysis validated the reliability of multimodal imaging for assessing the efficiency of stem cell delivery to tumors. The study involved the construction of a trimodal imaging method with two different labeling methods (direct labeling and reporter gene labeling) to provide a more comprehensive response to the in vivo delivery of stem cells that can be used to carry antitumor drugs to tumors.

Later, Tennstaedt et al. [78] applied ^19^F-MRI imaging to monitor the fate of stem cells transplanted into the striatum of the mouse brain, which, in addition to providing 3D cell localization, also quantified cells based on the signal-to-noise ratio response to cell density, avoiding the toxicity of SPECT imaging, which requires radioactive element labeling, to stem cells. In addition, BLI has provided information on cell viability and fluorescence signals in ex vivo fluorescence imaging to verify the results of in vivo imaging. The study revealed that different transplantation sites have different fates for stem cell survival and determined the effect of different environments in vivo on the survival rate and differentiation of transplanted stem cells, providing a reliable basis for improving the efficiency of cell therapy. In a study of stem cell therapy for myocardial infarction by Kim et al.'s team, multimodal imaging provided information on transplanted stem cells in vivo by BLI, and MRI showed viable cardiomyocytes in the infarcted region by the injection of a manganese-based contrast agent to assess the direct effect of stem cells on myocardial viability [54].

Although the combination of MRI and BLI provides information about cell function, localization, and quantification, the temporal resolution of MRI and BLI is low. In comparing the homing and migration ability of stem cells of different origins in vivo in real time, Oliveira et al. [79] combined two fluorophores, NIRF and rhodamine-B, with SPION for immediate imaging by NIR, compensating for the signal lag of MRI and BLI.

The random insertion of conventional viral vectors delivering transgenes increases the risk of altering the biological properties of stem cells, especially when multiple reporter genes are integrated into a single cell. Kelly et al. used CRISPR–Cas9 gene editing technology to knock in three reporter genes (for BLI, fluorescence imaging, and MRI) into AAVS1, the most commonly selected safe harbor site in targeted gene therapy, thereby enabling multimodal longitudinal in vivo imaging of cells and avoiding the expression of proto-oncogenes due to random integration. Rather than using the traditional mechanism of homologous targeted repair for gene integration, the authors combined the mechanism of homology-independent targeted integration (HITI), either in dividing or nondividing cells, to provide the efficiency of correct gene knock-in [80], laying the foundation for the clinical application of multimodal reporter genes.

However, when multiple reporter genes are integrated into a single cell, in addition to biosafety considerations, one should also consider whether it is possible to achieve the simultaneous expression of signals by all reporter genes to reach the minimum detection threshold for different imaging modalities. For example, when Parashurama et al. detected transplanted bone marrow-derived MSCs in a mouse myocardial infarction model, even though the transplanted cells simultaneously expressed luciferase 2 (Fluc2), EGFP, and the positron emission tomography (PET) reporter gene sr39ttk, the injected cells produced a PET signal that was well below the minimum detectable signal, and PET detection was therefore not possible [81].

*(3) MRI/Photoacoustic Multimodal Imaging*. Both BLI and MRI have relatively poor temporal resolution, while photoacoustic imaging provides excellent temporal resolution. Photoacoustic imaging can monitor the stem cell delivery process in real time, and MRI can provide clear anatomical information. This multimodal approach combines the advantages of photoacoustic temporal resolution and MRI anatomical resolution perfectly. For example, Kubelick and Emelianov used superparamagnetic Prussian blue nanocubes as a dual-mode contrast agent for MRI-photoacoustic imaging to label stem cells that were injected into the rat spinal cord, and thus, they achieved accurate localization of stem cell therapy under real-time guidance of photoacoustic imaging [82]. Additionally, MRI provided high-resolution anatomical information on the spinal cord to complement the information about the transplanted stem cells, enabling intraoperative and postoperative feedback regarding stem cell delivery for clinicians assessing the effect of stem cell therapy.

In monitoring transplanted stem cell populations in mouse myocardium, Lemaster et al. [23] used synthetic melanin nanoparticles loaded with gadolinium as a dual-mode contrast agent for MRI-photoacoustic imaging to label stem cells. In addition to combining the excellent temporal resolution of photoacoustic imaging with the anatomical resolution of MRI, synthetic melanin chelated with gadolinium had a 40-fold higher intensity of the photoacoustic signal compared to synthetic melanin alone, improving the sensitivity of photoacoustic detection. However, this labeling method does not allow long-term monitoring of the survival status of transplanted cells. Liu et al. established a multifunctional reporter gene system expressing tyrosinase for multimodal imaging of MRI, photoacoustic imaging, and PET. The expression of tyrosinase promotes the synthesis of the photoacoustic imaging reporter probe melanin, in addition to the combination of iron and the positron imaging agent 18F-5-fluoro-N-[2-(diethylamino)ethyl] picolinamide (18F-5-FPN) for MRI and PET, respectively. It also provides long-term monitoring of cell survival status and anatomical information for clear analysis [83]. The introduction of PET also compensates for the limitations of MRI-optical acoustic multimodal imaging methods that are not sensitive enough to detect a small number of cells.

## 3. Conclusions and Prospects

A variety of reliable methods for in vivo tracing of stem cells have been established in small animal models, and to minimize the limitations of each imaging technique, an increasing number of researchers are choosing multimodal imaging approaches with the help of appropriate cell labeling methods. There are different combinations of methods for different experimental purposes, and different cell labeling methods have different advantages and disadvantages. A combination of direct and indirect labeling can provide more complete information on stem cells in vivo.

Despite the progress made in multimodal imaging technology, significant challenges remain to be addressed in its clinical implementation. Improving imaging technology, developing safer and more versatile labels, and optimizing methods for multimodal image colocalization and registration are all crucial steps in this regard. In large animal models, MRI can overcome the problem of imaging depth, and MRI reporter genes behave similarly to BLI reporter genes; i.e., signals within the body indicate the survival status of stem cells within it. However, the current common MRI reporter gene FTH1 has low sensitivity in conventional T2WI sequences. As MRI technology is constantly updated, an increasing number of new techniques can sensitively detect reporter gene signals, such as magnetic SWI, a technique that responds to magnetic susceptibility differences between tissues, and quantitative susceptibility mapping (QSM), which can quantify iron content. It is conceivable that in the future, MRI will be able to provide not only cell localization and survival status but also quantitative cell analysis in large animal models.

To fully elucidate the functioning of stem cells in vivo at the cellular, subcellular, and protein levels, advancements in optical microscopy must be accompanied by developments in the safety, sensitivity, and pluripotency of probes.

Currently, the validation of in vivo imaging results in small animal models, such as stem cell migration localization and differentiation of stem cells in vivo, is mainly performed by presenting 2D planar images in tissue sections for histological and ex vivo fluorescence analysis. However, organisms are three-dimensional, and verifying the imaging results of stem cells in vivo is more accurate in a three-dimensional context. The internal structure of sample tissue after tissue section processing is easily destroyed, and reconstruction is time-consuming and inaccurate. By utilizing tissue transparency technology in conjunction with confocal, two-photon, and light-sheet microscopy, it is possible to achieve 3D imaging with high resolution, perform quantitative spatiotemporal analysis at the single-cell resolution level, and compare and register with MRI images. This approach builds the bridge between small animal models and larger animal as well as clinical studies.

At present, most studies of in vivo stem cell tracing are limited to basic research in small animal models, and the results of the above multimodal imaging studies are difficult to repeat in large animal models of preclinical experiments and clinical experiments, such as the use of BLI and fluorescence imaging, which are limited by the penetration depth. Therefore, the more accurate observation of stem cell fate in vivo through small animal models compensates for the limited results observed in large animal models or clinical experiments and reliably suggests the possible fate and effects of stem cells in humans. Apart from the challenge of achieving a breakthrough in cellular-level imaging technology within the human body, there are other difficulties to be considered, such as biosafety concerns associated with introducing various cellular labels. Moreover, the limited availability of FDA-approved contrast agents adds to this challenge. Therefore, exploring alternative imaging methods that do not require additional contrast agents, such as CEST and SHG imaging technology, holds promise for future clinical applications.

## Data Availability

The previously reported data were used to support this systematic review and are available at DOI. These prior studies are cited at relevant places within the text as references.
